# Tanshinone IIA attenuates the stemness of breast cancer cells via targeting the miR-125b/STARD13 axis

**DOI:** 10.1186/s40164-022-00255-4

**Published:** 2022-01-20

**Authors:** Xiaoman Li, Qi Jia, Yinyin Zhou, Xuan Jiang, Li Song, Yuanyuan Wu, Aiyun Wang, Wenxing Chen, Shijun Wang, Yin Lu

**Affiliations:** 1 Jiangsu Key Laboratory for Pharmacology and Safety Evaluation of Chinese Materia Medica, School of Pharmacy, Nanjing University of Chinese Medicine, Nanjing, 210023 China; 2 State Key Laboratory Cultivation Base for TCM Quality and Efficacy, Nanjing University of Chinese Medicine, Nanjing, 210023 People’s Republic of China; 3 Jiangsu Collaborative Innovation Center of Traditional Chinese Medicine (TCM) Prevention and Treatment of Tumor, Nanjing University of Chinese Medicine, Nanjing, 210023 People’s Republic of China; 4 College of Traditional Chinese Medicine, Shandong University of Traditional Chinese Medicine, Jinan, 250355 China

**Keywords:** Tanshinone IIA, miR-125b, STARD13, Stemness, Breast cancer

## Abstract

**Background:**

Tanshinone II A is an effective component extracted from Salvia miltiorrhiza and the roles of Tanshinone IIA in regulating the stemness of tumor cells remain unclear. This work aims to explore the roles and underlying mechanisms of Tanshinone IIA in breast cancer stemness.

**Methods:**

In vitro mammary spheroid formation, flow cytometry assay on CD24^−^/CD44^+^ sub-population, ALDH activity detection, cell viability assay and western blot analysis, and in vivo tumor-initiating analysis were performed to examine the effects of Tanshinone IIA on the stemness of breast cancer cells. MiRNAs-based transcriptome sequencing and data analysis, online dataset analysis, luciferase reporter assay combined with rescuing experiments were constructed to explore the underlying mechanisms.

**Results:**

Tanshinone IIA attenuated the stemness of breast cancer cells, evident by downregulating the expression of stemness markers, hindering the capacity of spheroid formation, decreasing the CD24^−^/CD44^+^ sub-population in a concentration-dependent manner and reducing the tumor-initiating ability of breast cancer cells. Additionally, Tanshinone IIA enhanced adriamycin sensitivity and attenuated adriamycin resistance of breast cancer cells. Combined with miRNAs-based transcriptome sequencing assay, it was found that Tanshinone IIA downregulated miR-125b level and upregulated its target gene STARD13 (StAR-related lipid transfer protein 13) level, thus inactivating the miR-125b/STARD13 axis, which had been previously confirmed to promote breast cancer progression. Notably, miR-125b overexpression enhanced the stemness of breast cancer cells, and miR-125b overexpression or STARD13 knockdown impaired the inhibitory effects of Tanshinone IIA on the stemness of breast cancer cells.

**Conclusions:**

Tanshinone IIA could attenuate the stemness of breast cancer cells via targeting the miR-125b/STARD13 axis.

**Supplementary Information:**

The online version contains supplementary material available at 10.1186/s40164-022-00255-4.

## Introduction

In women worldwide, breast cancer is the most common malignancy [1]. Specifically, its incidence ranks the first among female cancer [2]. Although there are various treatment methods in clinic, metastasis and drug resistance eventually occur, finally leading to the death of breast cancer patients [3]. Thus, it is still an urgent need to explore novel ways treating breast cancer metastasis and drug resistance.

According to cancer stem cells (CSCs) theory, a very small proportion of undifferentiated cells in tumor tissues has a strong capability to renew, divide, and constantly produce new tumor cells, called CSCs [4], which have been thought to be the root of tumor occurrence, recurrence, progression, drug resistance, and metastasis [5]. CSCs-targeted methods are becoming novel potential methods to treat cancer [6]. Tanshinone IIA is a fat-soluble component of traditional Chinese medicine danshen (salvia miltiorrhiza) [7]. The previous work has shown that Tanshinone IIA could ameliorate inflammation microenvironment in colorectal cancer via repressing miRNA-155 expression (8) and exert an anti-angiogenic effect in vascular endothelial cells via inhibiting the VEGF/VEGFR2 pathway [9]. Additionally, Tanshinone IIA induces the apoptosis and inhibits the stemness of human glioma stem cells [10]. And a recent study demonstrates that Tanshinone IIA restrains migratory and invasive ability of cervix carcinoma stem cells by suppressing the transcriptional activity of YAP (Yes-associated protein) [11]. We previously showed that cryptotanshinone, another component of danshen, can inhibit cell proliferation of melanoma cells [12], and mTORC1 pathway through activating the AMPK-TSC2 pathway [13] and dependent on ERα in breast cancer cells [14]. Recently, cryptotanshinone has been indicated to reduce the CSC-like traits of non-small cell lung cancer cells [15] and prostate CSCs [16]. However, the underlying roles and mechanism of Tanshinone IIA in modulating breast cancer stemness are still confusing.

MiRNAs (microRNAs) are small non-coding RNAs that could downregulate mRNA expression through a direct binding to their 3' untranslated region (3'UTR) [17]. MiRNAs regulate various aspects of cancer cells, like cell apoptosis, migration and autophagy [18, 19]. The study has previously revealed that miR-125b faciliates cell migratory ability breast cancer via targeting STARD13 (StAR-related lipid transfer protein 13) [20]. Reversely, we further showed that the 3'UTR of STARD13 mRNA reduces the migratory ability of breast cancer cells via regulating miR-125b activity [21, 22]. Importantly, our recent work demonstrates that the 3'UTR of STARD13 mRNA attenuates breast cancer stemness via hindering YAP/TAZ (transcriptional coactivator with PDZ-binding motif) activity through co-regulating Rho-GTPase/F-actin and Hippo signaling [23]. Notably, the previous work indicated that miR-125b could promote the dedifferentiation of acute myeloid leukemia cells [24]. These results promote us to further investigate if miR-125b could facilitate breast cancer stemness and if there is a drug that could attenuate breast cancer stemness via downregulating miR-125b level and thus inactivating the miR-125b/STARD13 axis.

Here, combined with the miRNA-based transcriptome sequencing, we found that Tanshinone IIA downregulated miR-125b level and upregulated its target gene STARD13 level in a manner depending on concentrations. Additionally, Tanshinone IIA attenuated breast cancer cells stemness, while miR-125b enhanced it. Furthermore, we indicated that Tanshinone IIA suppressed breast cancer cell stemness in an miR-125b/STARD13 axis-dependent manner. Our results revealed the critical roles and mechanisms of Tanshinone IIA in modulating breast cancer stemness, providing more evidences confirming Tanshinone IIA’s anti-tumor effects and indicating miR-125b as a potential target for targeting breast CSCs.

## Methods

### Cell culture and reagents

Human breast cancer cell lines MDA-MB-231, and MCF-7 were stored in our laboratory and short tandem repeat (STR) DNA profiling method was utilized to authenticate cell lines every 6 months. Adriamycin-resistant MCF-7 cells (MCF-7-Adr) were bought from KeyGen BioTECH (Nanjing, China) and the resistance index was evaluated before using for experiments. MDA-MB-231, MCF-7-Adr, and MCF-7 cells were cultured in the ways as described in our previous study [23]. Tanshinone IIA was purchased from Shanghai yuanye Bio-Technology and adriamycin was bought from Zhongda Hospital Southeast University.

### Quantitative real-time PCR (qRT-PCR)

Total RNA was extracted from cells using RNA isolater (Vazyme, Nanjing, China). Complementary DNA (cDNA) for mRNA or miRNA was then synthesized with HiScript RT SuperMix (Cat # R223-01, Vazyme). MiRNA/U6 snRNA (small nuclear RNA) RT primer mix and PCR specific primer set (GenePharma, China) were used to reversely synthesize cDNA for miRNAs, detect and quantify miRNAs expression, respectively. qRT-PCR was performed with qPCR SYBR® Green Master Mix (Vazyme) on Real-Time PCR system (Applied Biosystems, USA). U6 snRNA or GAPDH was used as an endogenous control for miRNA or mRNA, respectively. 2^−∆∆Ct^ method was carried out to measure the relative expression levels of mRNA or miRNA. The primer sequences were mentioned in our previous study [23].

### Western blot assay

This experiment was carried out as described in our previous study [25]. The antibodies’ detailed information in this work was listed as below: ALDH1A1 (Cat # 15,910–1-AP, Proteintech; Dilution rate: 1:1000), STARD13 (Cat # AP19692c, Abgent, Wuxi, China; Dilution rate: 1:1000), Oct3/4 (octamer-binding transcription factor 4) (Cat # wl01728, Wanleibio, Shenyang, China; Dilution rate: 1:2000), Ki67 (Cat # ab16667, Abcam; Dilution rate: 1:3500), Cleaved PARP (poly ADP-ribose polymerase) (Cat # ab32064, Abcam; Dilution rate: 1:2000), PARP (Cat # 13,371–1-AP, Proteintech; Dilution rate: 1:1500) and GAPDH (Cat # YFMA0037, YI FEI XUE Biotechnology, Nanjing, China; Dilution rate: 1:5000).

### Small interfering RNA (siRNA) synthesis, MiR-125b mimics, inhibitor, and transfection

Cells reaching 40%—60% cell confluency were transfected with 50 nM miR-125b inhibitor, mimics, and corresponding negative control (NC), siRNA against STARD13 (si-STARD13) and corresponding NC (si-NC). All of them were synthesized in Biomics Biotechnology (Nantong, China). Cells confluency of 80% was used for plasmid transfection. Transfection procedure was performed using jetPRIME (Polyplus Transfection, France). The sequences of siRNAs, miR-125b (inhibitor or mimics) and STARD13 overexpression vector (STARD13) were mentioned in our previous studies [20–23, 26].

### Flow cytometric assay

Different concentrations (2.5 μM, 5 μM, 10 μM, and 20 μM) of Tanshinone IIA were added into cells plus transfection of miR-125b mimics (50 nM) or si-STARD13 (50 nM). After 48 h, CD24^−^/CD44^+^ sub-population was measured on a C6 flow cytometer (BD Biosciences) following our previously described procedure [23]. And cell cycle distribution was analyzed using a Cell-Cycle Detection Kit (Cat # KGA511, KeyGen BioTECH) following the manufacturer’s recommendation.

### Cell spheroid formation assay

MammoCult medium (Stem Cell Technologies, Vancouver, Canada) were utilized for culturing cell spheroids supplemented with MammoCult Proliferation Supplements (Stem Cell Technologies) as well as 0.48 μg/mL Hydrocortisone (STEMCELL Technologies), 4 μg/mL Heparin (STEMCELL Technologies). And cells were plated in ultra-low attachment 24-well plates (Corning, USA) at a 1000 cells/well density. After culturing for 9 days, cell spheroids were photographed and counted.

### Cell viability assay

24 h, 48 h, and 72 h later, cell viability was measured utlizing MTT staining for cells cultured in 96-well plates and treated with adriamycin as well as Tanshinone IIA. Briefly, MTT (5 mg/ml) was added into cells for staining and the resulted formazan crystals was dissolved utilizing 200 μl DMSO, and the absorbance was detected on a spectrophotometer (BIO-Tek) at a 570 nm of wavelength.

### ALDH (Aldehyde Dehydrogenase) activity assay

Cells were transfected or treated for 48 h, ALDH activity was analyzed utilizing Aldehyde Dehydrogenase Activity Colorimetric Assay Kit (Cat # MAK082, Merck) following the standard procedure.

### Luciferase reporter assay

The luciferase reporter vector containing STARD13 3’UTR (Luc-STARD13-3'UTR) was restored in our laboratory [21–23, 26, 27]. MiR-125b, Luc-STARD13-3'UTR, and β-gal were co-transfected into cells, and then treated with Tanshinone IIA. The luciferase activity was measured 72 h later as described in our previous study [23].

### RNA sequencing, data and online dataset analysis

MiRNAs-based transcriptome sequencing and analysis were carried out by Novogene (Beijing, China). Gene Expression Omnibus (GEO) database was utilized to save the data, denote as **GSE156155**. Kaplan – Meier (KM) Plotter analysis was carried out through the online dataset (GSE40267) (http://kmplot.com/analysis/index.php?p=background). OncomiR: Database Search (http://www.oncomir.org) was used for analyzing the correlation between miRNAs and clinical parameters.

### In vivo tumor-initiating ability and immunohistochemical (IHC) assays

This experiment was detailed in our previous work [28]. In brief, six-week-old female athymic BALB/c nude mice were bought from GemPharmatech (Nanjing, China), fed, and housed under standard pathogen-free conditions. The tumor-initiating ability of cells was evaluated utilizing tumor-limiting dilution assay. For analyzing the effects of Tanshinone IIA, 1 × 10^7^, 1 × 10^6^, 1 × 10^5^ MDA-MB-231 cells were pre-cultured with Tanshinone IIA (10 μM) for 72 h or not and then orthotopically implanted in mice. For determining the effects of miR-125b on the tumor-initiating ability of breast cancer cells and whether miR-125b is involved in Tanshinone IIA-mediated effects, MCF-7 cells were infected with control lentivirus (vector) or miR-125b with GFP labeling (Genepharma, Shanghai, China) and screened for stable-infected cell lines using puromycin (2 μg/ml, Sigma). Then MCF-7 cells with vector or miR-125b stable overexpression were pre-cultured with or without Tanshinone IIA (10 μM) for 72 h and then implanted in mice orthotopically. On day 14, mice were subjected to optical in vivo imaging and euthanized. Tumor tissues were collected, weighed and used for IHC analysis on stemness marker expression. An ELDA tool (http://bioinf.wehi.edu.au/software/elda/) was utilized to calculate the stem cell frequencies [29]. For analyzing the correlation between Tanshinone IIA treatment and survival of mice, 1 × 10^7^ MDA-MB-231 cells pre-treated with Tanshinone IIA or not were orthotopically implanted in mice. Then mice weight was measured for two weeks every three days, and the mice survival was observed for the long time.

All animal experiments were constructed following the approval of the Ethics Committee for Animal Experimentation of Nanjing University of Chinese Medicine.

### In vivo EdU incorporation assay

For the EdU incorporation experiment, mice bearing tumors formed by MDA-MB-231 cells with or without Tanshinone IIA pretreatment were received intraperitoneal injection of EdU (Beyotime, Shanghai, China) at a dose of 5 mg/kg body weight every 2 h for three times. 24 h later, mice were euthanized, tumors were removed, and fixed in 4% paraformaldehyde at 4℃ overnight. The tumors were then sectioned at 3 μm from the paraffin-embedded tissue blocks, stained with EdU. The fluorescence intensity of EdU-labeled cells was quantified using fluorescence microscope.

### Statistical analysis

At least three independent experiments (n ≥ 3) were performed to obtain data and data were denoted as the mean ± SD (standard deviation). Student’s t test excepting for qRT-PCR was used for statistical analysis. One-way analysis of variance (ANOVA) was utilized to analyze data from the qRT-PCR. ANOVA with the Tukey–Kramer post-test was utilized to assay the differences between the groups. Data were considered to be statistically significant with *p < 0.05 or less.

## Results

### Tanshinone IIA attenuates breast cancer stemness

**Fig. 1:**
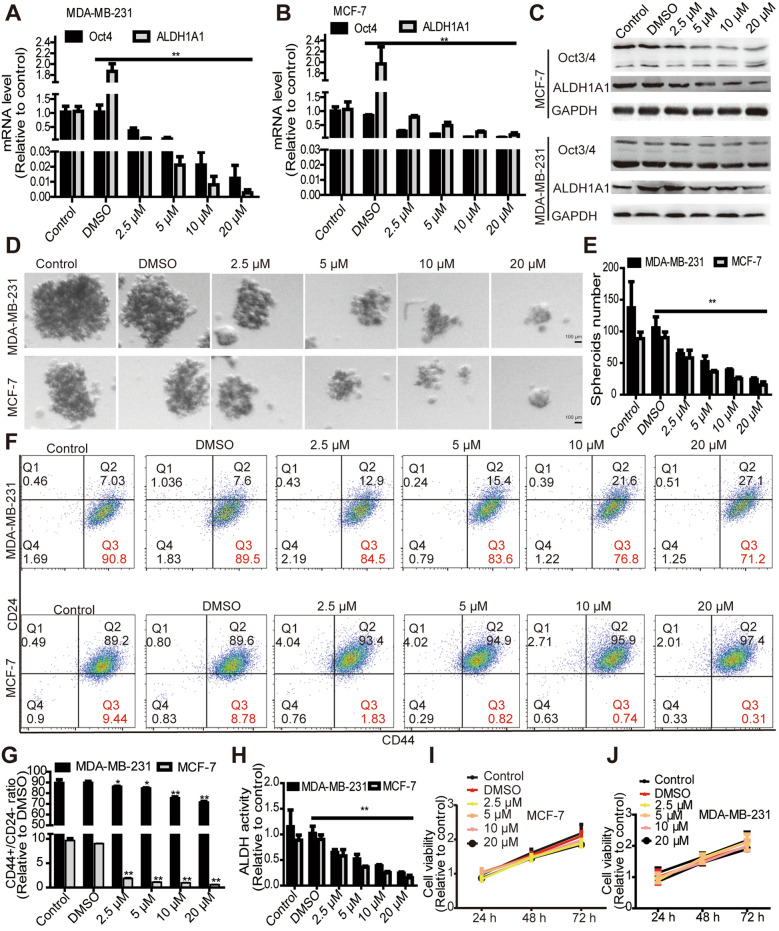
Tanshinone IIA attenuates breast cancer stemness in vitro. **A**, **B** qRT-PCR was utilized to examine the mRNA levels of stemness markers (Oct4 and ALDH1A1) in Tanshinone IIA-treated breast cancer cells. **C** Tanshinone IIA-treated breast cancer cells were followed by examination of stemenss markers (Oct3/4 and ALDH1A1) protein levels through western blot. **D**, **E** Tanshinone IIA-mediated effects on spheroid formation were evaluated by measuring the spheroid size (**D**) and number (**E**). **F**, **G** Representative FACS profile of breast cancer cells were detected with CD44^+ ^and CD24^−^ markers and quantified. **H** ALDH activity was measured in Tanshinone IIA-treated breast cancer cells. **I**, **J** Cell viability was assessed in the cells described in **A**. **p < 0.01 vs. Control, n = 3

Firstly, we detected the effects of Tanshinone IIA on stemness markers (Oct3/4 and ALDH1A1) expression. Western blot and qRT-PCR analysis showed that Tanshinone IIA decreased Oct3/4 and ALDH1A1 expression in breast cancer cells (Fig. 1A–C). Additionally, Tanshinone IIA attenuated cell spheroid-formation capacity, evidenced by the decreased spheroid number and size (Fig. 1D, E). Furthermore, Tanshinone IIA reduced the CD44^+^/CD24^−^ sub-population, which have been identified as the cell sub-population with CSC-like traits [30] (Fig. 1F, G). Moreover, ALDH activity was significantly reduced by Tanshinone IIA (Fig. 1H). Notably, Tanshinone IIA had little effects on cell viability at these concentrations (except for 20 μM) (Fig. 1I, J). What’s more, we detected the effects of Tanshinone IIA on the cell cycle progression of breast cancer cells, and viability of non-malignant MCF10A cells, and revealed that Tanshinone IIA held no effect on the cell cycle progression of MCF-7 cells (Additional file 1: Fig. S1A) and viability of MCF-10A cells (Additional file 1:Fig. S1C), while it arrested cell cycle in G1 phase in MDA-MB-231 cells (Additional file 1: Fig. S1B).

**Fig. 2:**
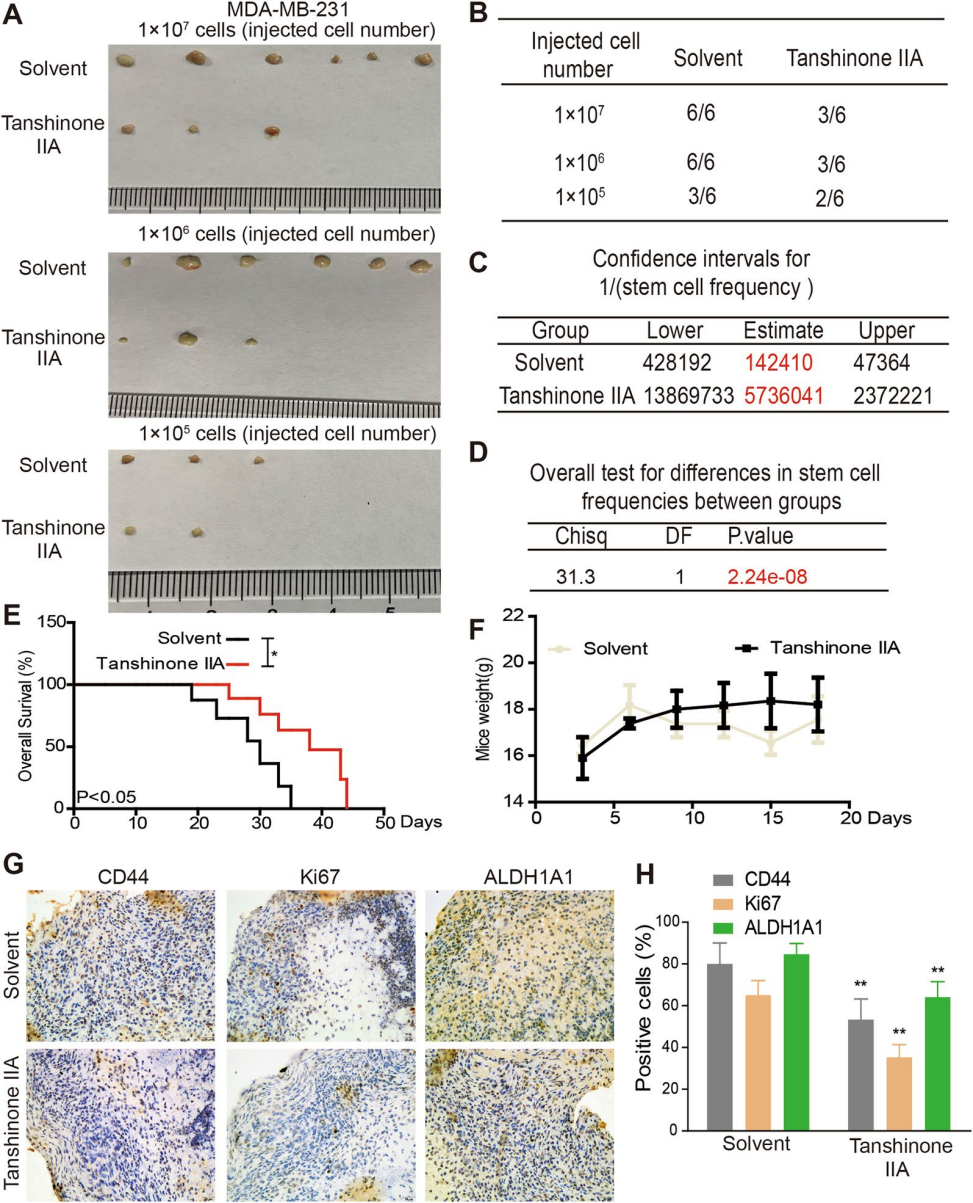
Tanshinone IIA attenuates breast cancer stemness in vivo. **A** The images of tumors derived from Tanshinone IIA (10 μM)-pretreated MDA-MB-231 cells or not. **B** The tumor-formation rate of **A**-described cells. **C**, **D** The confidence intervals for 1/(stem cell frequency) and differences between groups were calculated using the online ELDA analysis tool. **E** The survival of mice bearing tumors was determined. **F** The mice weight was measured at different time-points. **G**, **H** Stemness markers (CD44, ALDH1A1, Ki67) protein levels were evaluated in tumors described in **A** at the same cell concentration via IHC analysis. **p < 0.01 vs. Control, n = 3

The tumor-initiating ability was further determined to confirm the impacts on breast cancer stemness. As Fig. 2A, B results indicated that, Tanshinone IIA decreased the tumor-formation ratio of MDA-MB-231 cells, and ELDA analysis revealed the stem cell frequency was significantly reduced by Tanshinone IIA (Fig. 2C, D). Additionally, the survival of the mice bearing tumors was prolonged by Tanshinone IIA (Fig. 2E), and Tanshinone IIA had no toxicology, but exhibited a certain degree of protection by detecting mice weight although without significance (Fig. 2F). Furthermore, IHC (Immunohistochemistry) analysis also indicated that stemness markers (CD44, ALDH1A1, Ki67) expression was reduced in tumors formed by Tanshinone IIA pre-incubated MDA-MB-231 cells (Fig. 2G, H). Consistently, EdU incorporation assay revealed that the EdU-positive cells were significantly reduced in tumors by Tanshinone IIA pre-treatment (Additional file 1: Fig. S1D). These results prompt that Tanshinone IIA could attenuate breast cancer stemness.

### MiR-125b/STARD13 axis is a potential target of Tanshinone IIA

**Fig. 3:**
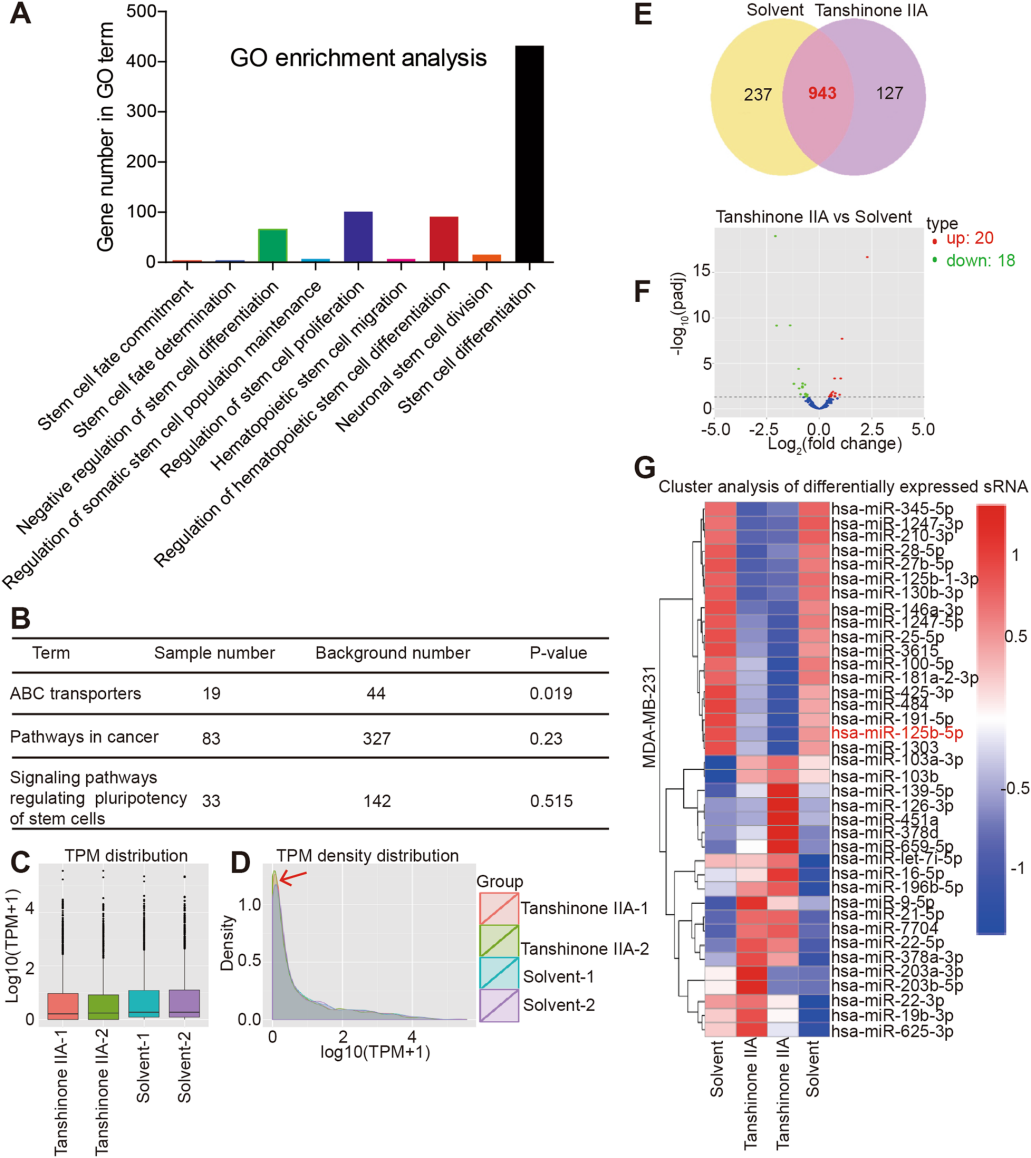
MiR-125b is involved in Tanshinone IIA-mediated effects in breast cancer cells. **A** Gene Ontology (GO) enrichment analysis based on miRNA-sequencing dataset in Tanshinone IIA-treated MDA-MB-231 cells or not. **B** KEGG analysis based on miRNA-sequencing dataset in Tanshinone IIA-treated MDA-MB-231 cells or not. **C** MiRNA TPM distribution based on miRNA-sequencing dataset in Tanshinone IIA-treated MDA-MB-231 cells or not. **D** MiRNA density distribution based on miRNA-sequencing dataset in Tanshinone IIA-treated MDA-MB-231 cells or not. **E** Venn diagram of miRNAs based on miRNA-sequencing dataset in MDA-MB-231 cells treated with Tanshinone IIA or not. **F** Volcano map miRNAs based on miRNA-sequencing dataset in Tanshinone IIA-treated MDA-MB-231 cells or not. **G** Cluster analysis of differentially expressed miRNAs in Tanshinone IIA-treated MDA-MB-231 cells or not

**Fig. 4:**
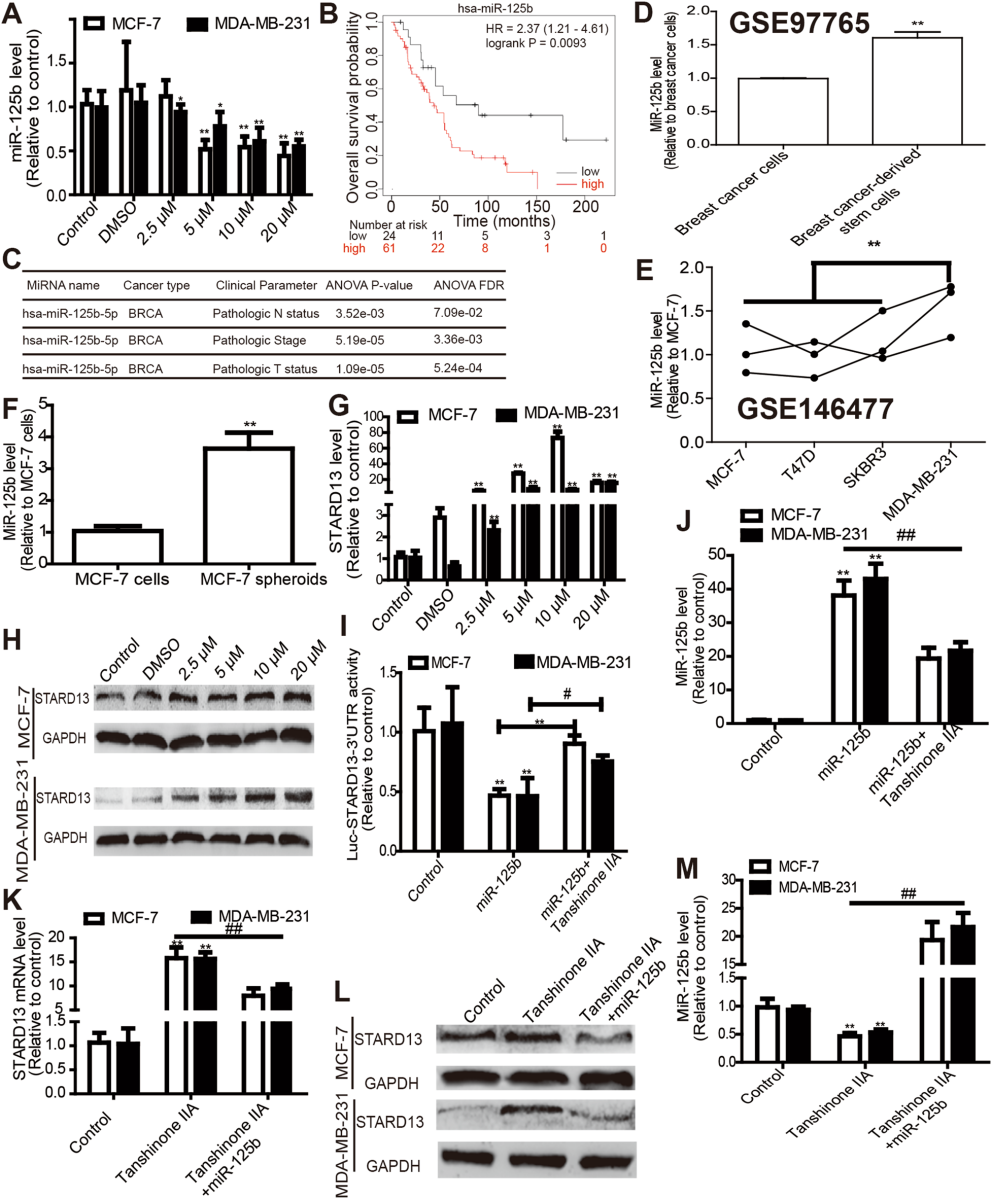
MiR-125b/STARD13 axis is a potential target of Tanshinone IIA. **A** MiR-125b level was measured in breast cancer cells treated with different concentration of Tanshinone IIA as indicated. **B** The correlation between the overall survival probability of breast cancer patients and miR-125b was examined through Kaplan – Meier (KM) Plotter analysis. **C** The correlation between the pathologic status of breast cancer patients and miR-125b level was examined through OncomiR analysis. **D** MiR-125b level was detected in breast cancer-derived stem cells and breast cancer cells via analyzing the GEO dataset (GSE97765). **E** MiR-125b level was detected in different types of breast cancer cells via analyzing the GEO dataset (GSE146477). **F** MiR-125b level was examined in MCF-7 spheroids and cells. **G** and **H** Expression of STARD13, a target of Tanshinone IIA was determined in cells depicted in **A**. **I** Luciferase activity of Luc-STARD13-3’UTR was examined in breast cancer cells with miR-125b overexpression plus Tanshinone IIA (10 μM). **J** MiR-125b level was evaluated in (I)-described cells. **K**, **L** STARD13 expression was examined in breast cancer cells with Tanshinone IIA (10 μM) as well as miR-125b overexpression or not. **M** MiR-125b level was detected in the cells depicted in **F**. n = 3, *p < 0.05, **p < 0. 01 vs. Control, ^##^p < 0. 01 vs. Tanshinone IIA. For transfection experiments, control groups were transfected with miR-125b mimics NC

Then we wonder whether miRNAs are involved in Tanshinone IIA-mediated effects on breast cancer cell stemness as miRNAs’ critical roles in tumor progression [31]. MiRNAs-based transcriptome-sequencing was constructed in Tanshinone IIA-treated MDA-MB-231 cells or not. Gene Ontology (GO) enrichment analysis revealed that Tanshinone IIA was involved in the stem cell fate, proliferation, migration, differentiation and division (Fig. 3A). Kyoto Encyclopedia of Genes and Genomes (KEGG) analysis indicated that Tanshinone IIA could regulate the pathways of *ABC transporters*, *Pathways in cancer* and *Signaling pathways regulating pluripotency of stem cells* (Fig. 3B). Importantly, the miRNA TPM (Transcripts PerKilobase Million) distribution (Fig. 3C) and density distribution (Fig. 3D) were different between Tanshinone IIA and solvent groups. These results further confirm that Tanshinone IIA could regulate breast cancer stemness, in which miRNAs are involved. We noted that there were total 943 miRNAs in MDA-231 cells with or without Tanshinone IIA treatment (Fig. 3E), and 20 miRNAs were upregulated and 18 miRNAs were downregulated in Tanshinone IIA-treated MDA-MB-231 cells (Fig. 3F), among which miR-125b-5p caught our attention as it has been confirmed to increase metastatic ability of breast cancer by us [21, 22, 25] (Fig. 3G). Consistently, qRT-PCR assay indicated that Tanshinone IIA decreased miR-125b level (Fig. 4A). Notably, KM-plotter analysis revealed that a higher level of miR-125b expression predicted a shorter overall survival of breast cancer patients (Fig. 4B). Moreover, online dataset analysis (OncomiR: Database search) indicated that miR-125b level is related to the pathologic status of breast invasive carcinoma (BRCA) (Fig. 4C), and GEO (GENE EXPRESSION OMNIBUS, GSE146447, GSE97765) analysis demonstrated that miR-125b level was remarkably upregulated in CSCs sorted from breast cancer cells (Fig. 4D) and triple negative breast cancer cells which has been proved to hold a stronger stemness than other types of breast cancer cells [23] (Fig. 4E). Studies have confirmed that CSCs are mostly enriched in non-adherent spheroids [32, 33]. Then miR-125b level was measured in MCF-7 spheroids and cells, and we found that MCF-7 spheroids exhibited a higher miR-125b level compared to that of cells (Fig. 4F). These results hint that miR-125b may be a critical contributor to breast cancer stemness.

We and others have established the promoting effects of miR-125b/STARD13 axis in the progression of breast cancer [20–23, 26] and that STARD13 is critical for suppressing breast cancer stemness [23]. Thus, we speculated that Tanshinone IIA attenuates breast cancer stemness through this miR-125b/STADR13 axis. Firstly, we investigated if Tanshinone IIA could inactivate this critical axis. As expected, western blot and qRT-PCR results indicated that Tanshinone IIA increased STADR13 expression (Fig. 4G, H). In addition, luciferase reporter analysis demonstrated that Tanshinone IIA attenuated miR-125b-mediated inhibition on Luc-STARD13-3'UTR activity (Fig. 4I). MiR-125b level was indeed remarkably upregulated by miR-125b transfection, which was attenuated by Tanshinone IIA treatment (Fig. 4J). Notably, transfection of miR-125b mimics impaired the promoting effects of Tanshinone IIA on STARD13 expression (Fig. 4K, L). QRT-PCR analysis confirmed miR-125b mimics’ transfection efficiency (Fig. 4M). These results indicate that Tanshinone IIA could indeed attenuate miR-125b-mediated inhibition on STARD13 expression in breast cancer cells.

### MiR-125b/STARD13 enhances breast cancer stemness

**Fig. 5:**
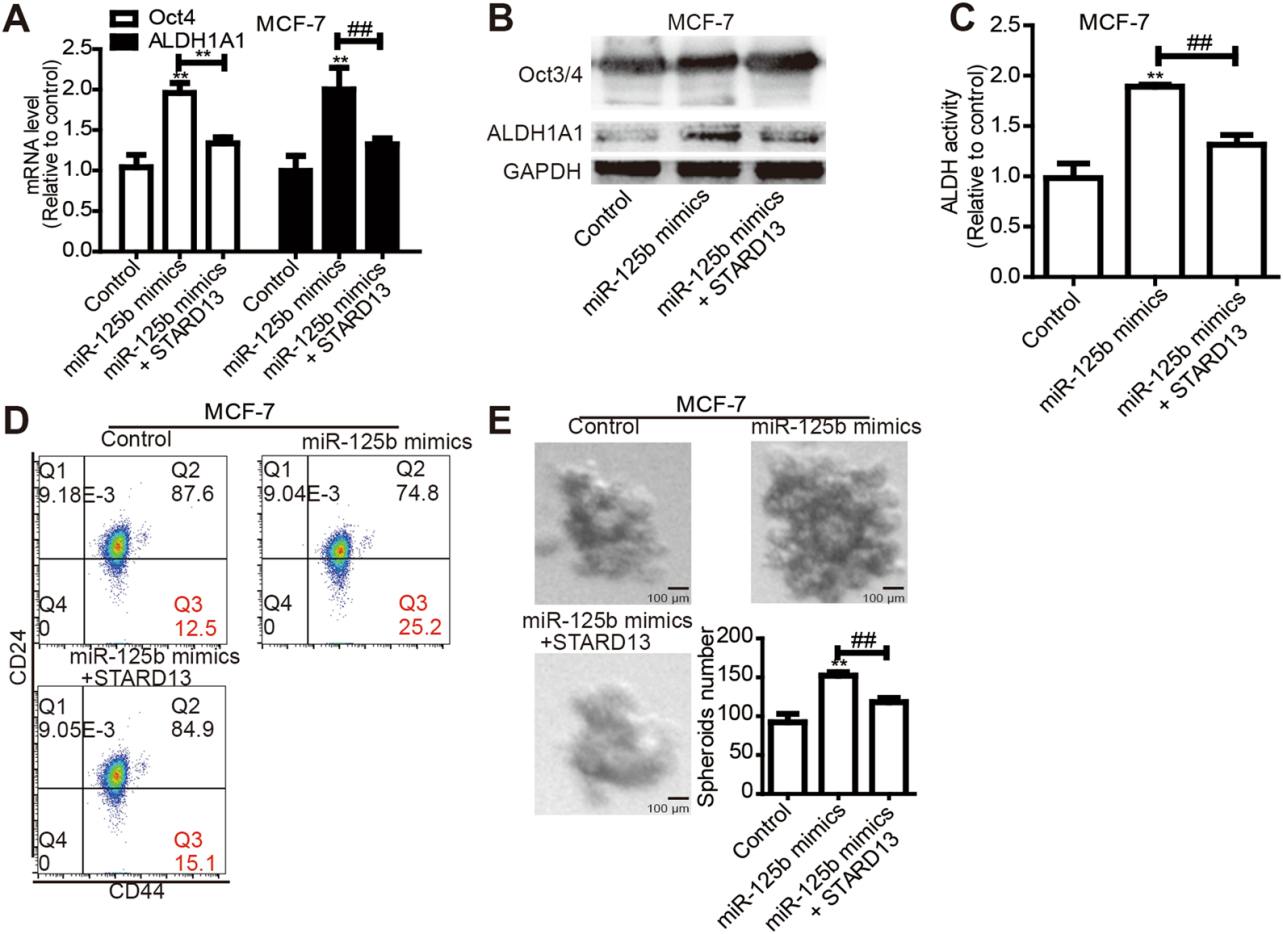
MiR-125b/STARD13 enhances MCF-7 cell stemness. **A**, **B** Expression levels of stemness markers (ALDH1A1and Oct3/4) were measured in miR-125b overexpressed-MCF-7 cells with overexpressing STARD13 3’UTR or not. **C** ALDH activity was detected in (A)-depicted cells. **D** Representative FACS profile of cells described in **A** were denoted with CD44 + and CD24 − markers. **E** The capacity of spheroid formation was determined in **A**-depicted cells. **p < 0. 01 vs. Control, ^##^p < 0. 01 vs. miR-125b mimics, n = 3. For transfection experiments, control groups were transfected with miR-125b mimics NC

Then we speculated if miR-125b could exert opposite effects through targeting STARD13 3'UTR. Owing to the relative stronger stemness of MDA-MB-231 and weaker stemness of MCF-7 cells [34, 35], miR-125b inhibitor was chosen tobe transfected into MDA-MB-231 cells, and miR-125b mimics into MCF-7 cells, respectively. As expected, miR-125b overexpression enhanced MCF-7 cell stemness, shown by the increasing of Oct3/4 and ALDH1A1 expression (Fig. 5A, B), ALDH activity (Fig. 5C), CD44^+^/CD24^−^ sub-population (Fig. 5D) and cell spheroid-formation capability (Fig. 5E); these effects were partially abrogated by overexpressing STARD13. Additionally, miR-125b inhibitor transfection attenuated MDA-MB-231 cell stemness, which was rescued by knocking down STARD13 as well (Additional file 2: Fig. S2).

### Tanshinone IIA attenuates breast cancer stemness partly dependent on miR-125b/STARD13 axis

**Fig. 6:**
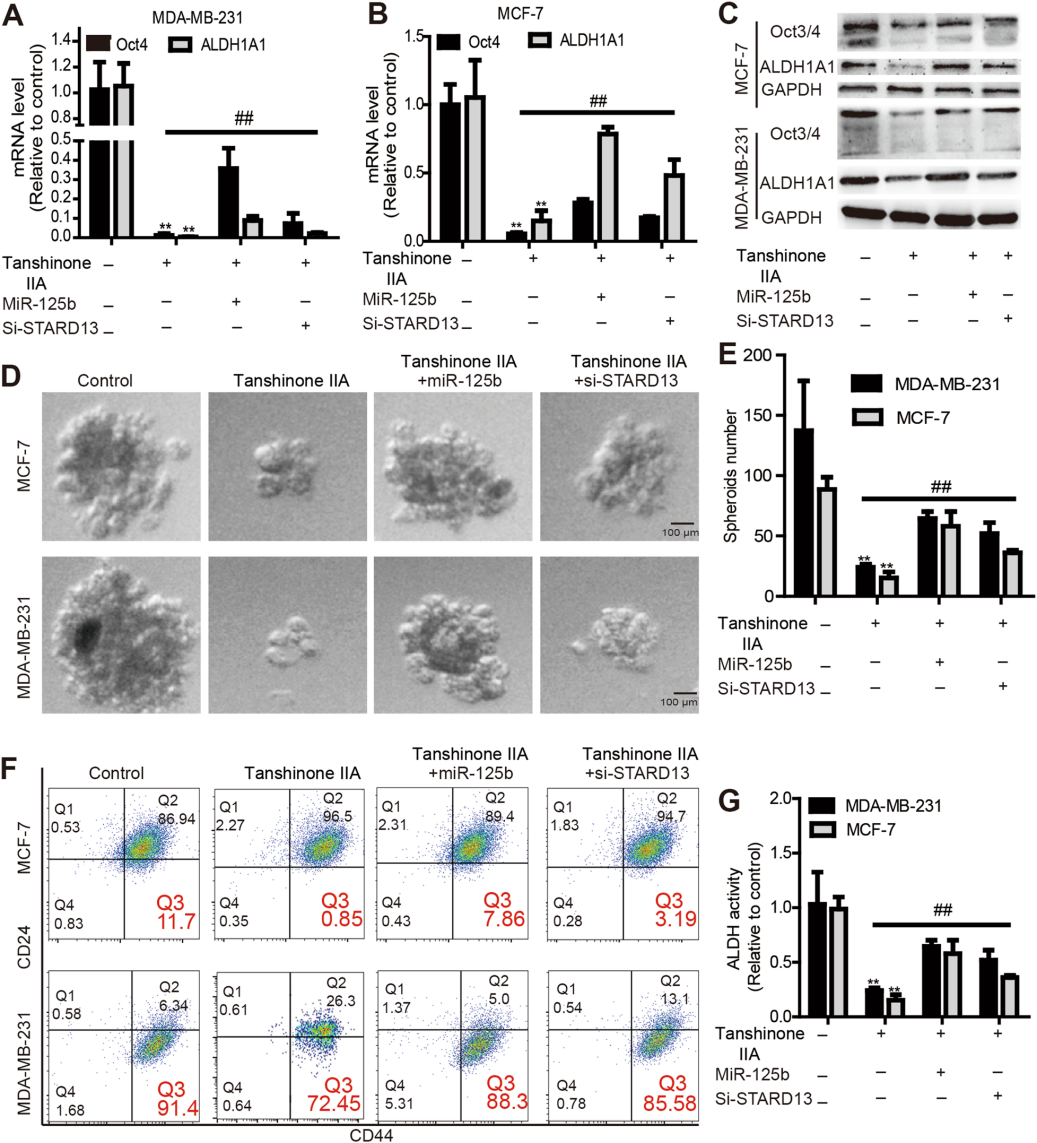
Tanshinone IIA attenuates breast cancer stemness partly dependent on miR-125b/STARD13 axis. **A**, **B** Breast cancer cells treated with Tanshinone IIA (10 μM) plus miR-125b overexpression or STARD13 knockdown and then subjected to qRT-PCR assay to examine stemness markers (Oct4 and ALDH1A1) mRNA levels. **C** Stemness markers (Oct3/4 and ALDH1A1) protein levels were evaluated in **A**-depicted cells. **D**, **E** Spheroid formation capacity was evaluated in **A**-depicted cells. **F** Representative FACS profile of **A**-depicted cells were denoted with CD24^−^ and CD44^+^ markers. **G** ALDH activity was measured in cells depicted in **A**. n = 3, **p < 0.01 vs. Control, ^##^p < 0. 01 vs. Tanshinone IIA. For transfection experiments, control groups were transfected with NC

**Fig. 7:**
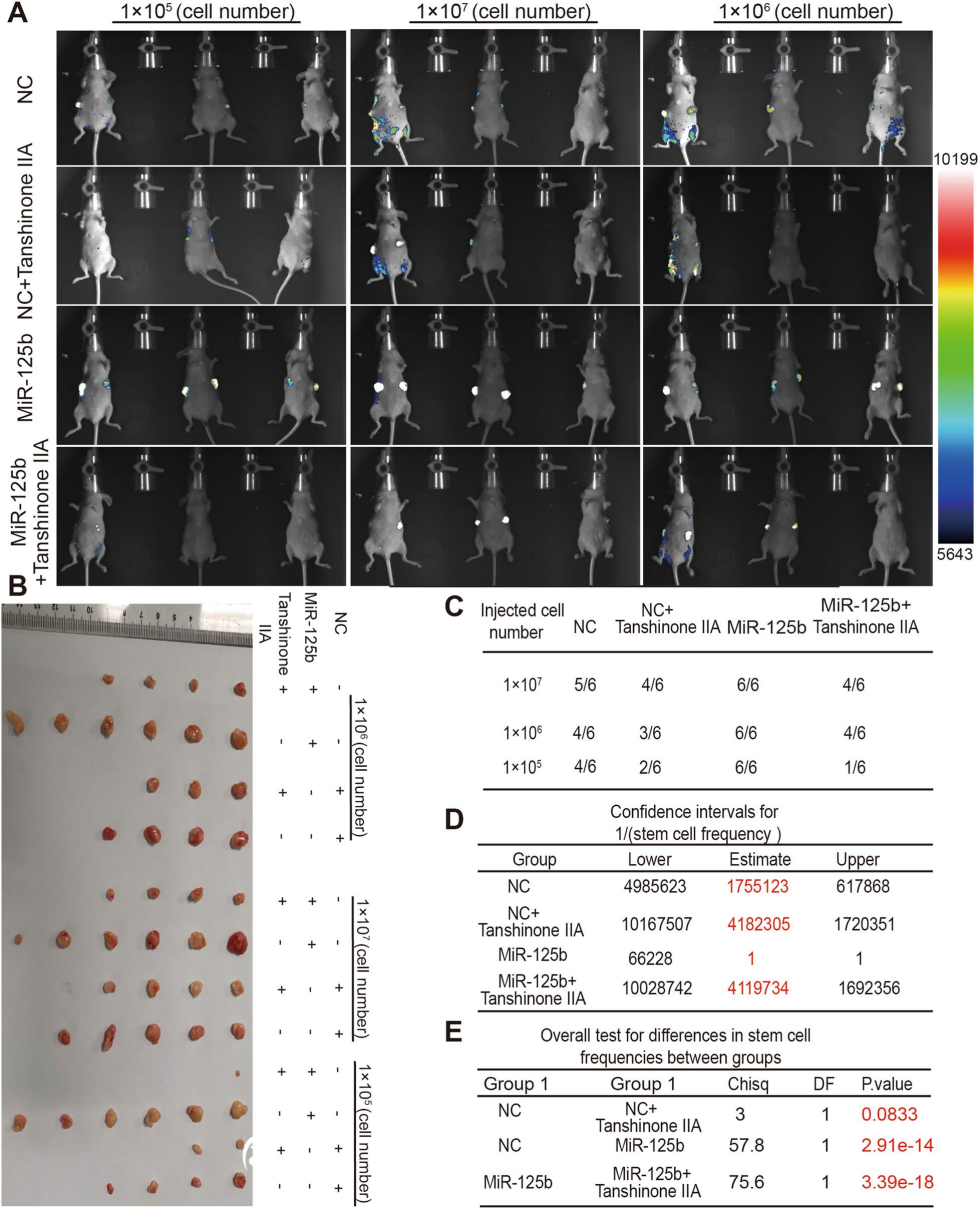
Tanshinone IIA attenuates breast cancer stemness partly dependent on miR-125b in vivo. **A** Mice inoculated with different cells as indicated were subjected to optical in vivo imaging and euthanized. **B** Tumor tissues were collected from mice depicted in **A**. **C**–**E** The tumor-formation rate, confidence intervals for 1/(stem cell frequency) and differences between groups of cells described in **A** were calculated using the online ELDA analysis tool

We then explored whether the miR-125b/STARD13 axis is responsible for the inhibition of Tanshinone IIA on breast cancer stemness. As exhibited in Fig. 6A–C, Tanshinone IIA-mediated downregulation of Oct3/4 and ALDH1A1 expression was partially reversed by overexpressing miR-125b or knocking down STARD13. Additionally, miR-125b overexpression or STARD13 knockdown attenuated Tanshinone IIA-mediated inhibition on cell spheroid formation (Fig. 6D, E) and CD44^+^/CD24^−^ sub-population (Fig. 6F). Furthermore, the decrease of ALDH activity induced by Tanshinone IIA was abrogated by overexpressing miR-125b or knocking down STARD13 (Fig. 6G). Additionally, we constructed miR-125b-stable overexpressed MCF-7 cells by lentivirus infection and pre-incubated with Tanshinone IIA or not, which were subjected to tumor-initiating ability analysis. It was found that the tumor-initiating ability was indeed promoted by overexpressing miR-125b, which was rescued by Tanshinone IIA treatment (Fig. 7A–E). A consistent result was obtained upon detecting the stemness marker expression in tumors (Additional file 3: Fig. S3). These results suggest that Tanshinone IIA attenuates breast cancer stemness at least through the miR-125b/STARD13 axis.

### Tanshinone IIA positively regulates adriamycin sensitivity by suppressing breast cancer cell stemness

**Fig. 8:**
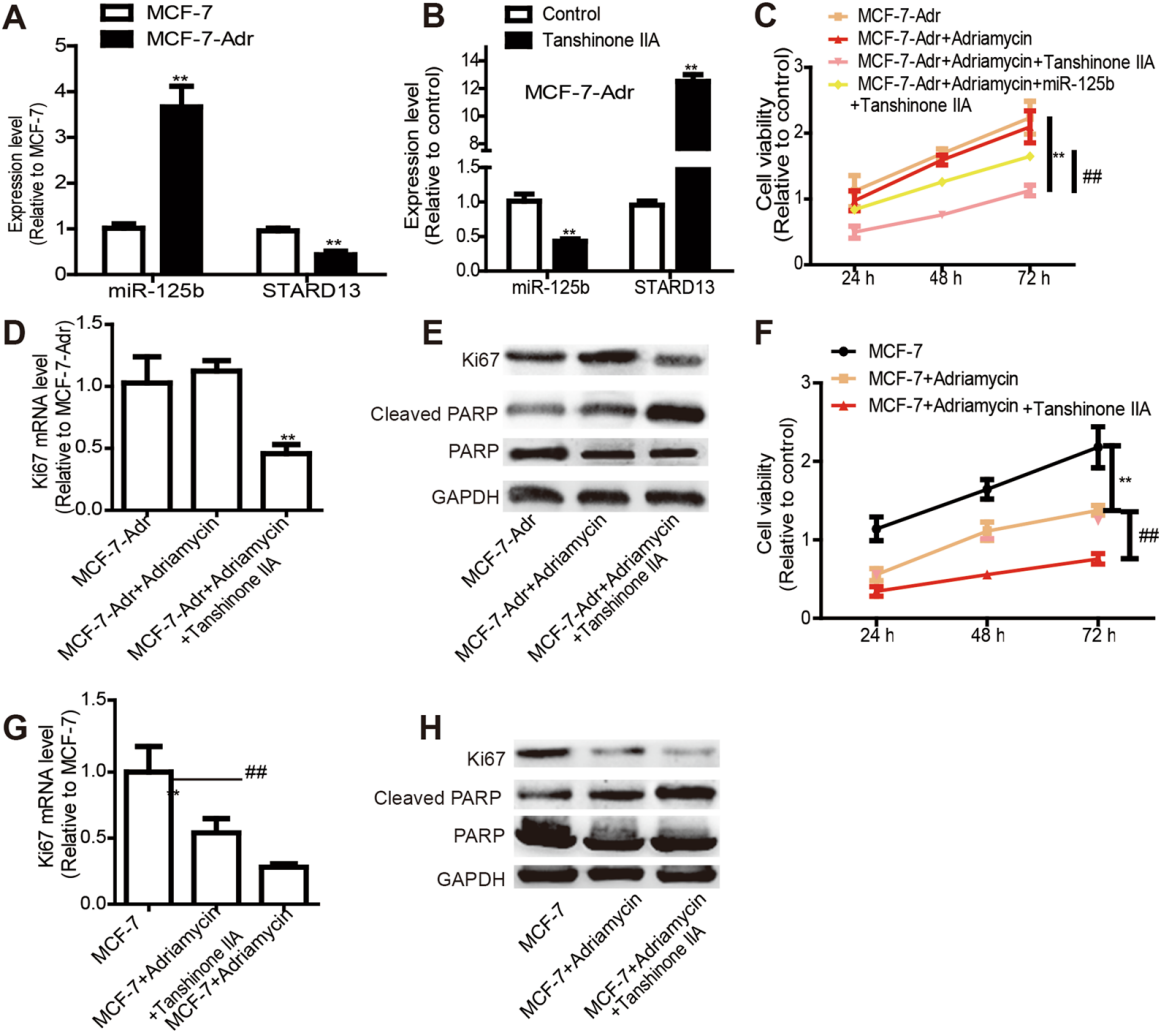
Tanshinone IIA positively regulates adriamycin sensitivity by suppressing breast cancer stemness. **A** MiR-125b and STARD13 levels were detected in MCF-7-Adr and MCF-7 cells. **B** MiR-125b and STARD13 levels were examined in Tanshinone IIA-MCF-7-Adr cells or not. **C** MCF-7-Adr cells were treated with adriamycin (2 μM) as well as Tanshinone IIA (10 μM), after 24 h, 48 h and 72 h, cell viability was evaluated by MTT assay. **D** Ki67 mRNA level was determined in cells depicted in **C** with 48 h treatment. **E** Protein levels of Ki67 and apoptotic executor (Cleaved PARP) were examined in **D**-described cells. **F** Cell viability was evaluated in MCF-7 cells with adriamycin (2 μM) as well as Tanshinone IIA (10 μM) treatment or not. **G** Ki67 mRNA level was measured in **F**-depicted cells. **H** Protein levels of Ki67 and apoptotic executor (Cleaved PARP) were examined in **E**-depicted cells. **p < 0.01 vs. Control, ^##^p < 0. 01 vs. MCF-7-Adr + Adriamycin + Tanshinone IIA group, n = 3

Since drug resistance can be led by tumor cell stemness [36, 37], we investigated if Tanshinone IIA could attenuate adriamycin resistance. Firstly, we detected miR-125b and STARD13 levels in MCF-7-Adr and parental MCF-7 cells, and identified that MCF-7-Adr cells exhibited a higher miR-125b level, while STARD13 exhibited an opposite effect (Fig. 8A). Consistently, miR-125b level was downregulated by Tanshinone IIA treatment, whereas STARD13 expression was increased by Tanshinone IIA treatment in MCF-7-Adr cells (Fig. 8B). In addition, Tanshinone IIA attenuated the adriamycin resistance in MCF-7-Adr cells, which was rescued by prior transfection of miR-125b mimics to Tanshinone IIA treatment (Fig. 8C). Furthermore, the expression of proliferation marker (Ki67) and apoptotic executor (Cleaved PARP) was decreased or increased by Tanshinone IIA plus adriamycin treatment, respectively (Fig. 8D, E). Moreover, Tanshinone IIA enhanced adriamycin sensitivity of MCF-7 cells, characterized as the additive effects on cell viability (Fig. 8F), the decreased Ki67 expression (Fig. 8G) and increased Cleaved PARP expression (Fig. 8H). Indeed, Tanshinone IIA decreased MCF-7-Adr cell stemness (Additional file 4: Fig. S4). As a result, our results demonstrate that Tanshinone IIA could positively regulate adriamycin sensitivity by suppressing breast cancer stemness.

## Discussion

Self-renewing and differentiation of breast CSCs contribute to the occurrence and recurrence of breast cancer, which is involved in breast cancer invasion and metastasis and the treatment resistance to radiotherapy and chemotherapy [36]. Thus, targeted therapy for this particular subgroup is likely to eradicate the tumor and prevent recurrence, thereby improving the prognosis of patients. Currently, we found that Tanshinone IIA attenuated breast cancer stemness. Mechanistically, the miR-125b/STARD13 axis plays an essential role in Tanshinone IIA-mediated suppressive effects. From our own perspectives, this study firstly shows the impairment of Tanshinone IIA on drug resistance, stemness, and sensitivity of breast cancer cells. Additionally, miR-125b effects in breast cancer stemness are firstly revealed in this work.

MiRNAs have been confirmed to be involved in CSC progression and are regarded as potential targets for breast cancers [38]. For example, miR-200c delivered by solid lipid nanoparticles attenuates the chemoresistance of breast CSCs [39]; miR-206 attenuates the migratory ability and stemness of breast cancer by targeting MKL1 (Myocardin-Like Protein 1) /IL11 (Interleukin11) signaling [40], and we recently indicated that miR-873 attenuated breast cancer stemness by directly targeting PD-L1 [41]. Dysregulated miR-125b expression holds various effects on tumor progression. However, miR-125b plays different or opposite effects in different cancers or tissues by targeting different transcripts. A recent study indicates that miR-125b inhibits osteosarcoma cell progression through MAPK-STAT3 signaling [42]. However, our previous studies show that miR-125b was highly expressed in lymph node metastatic breast cancer [21] and enhanced the metastatic ability of breast cancer cells [20]. Since CSCs can result in tumor metastasis, we wonder whether miR-125b-mediated promoting effects on breast cancer metastasis are due to its facilitating effects on breast cancer stemness, this should be explored in the future. The miR-125b/STARD13 regulatory axis has been confirmed in our previous studies [20–22, 26], and we indicate that STARD13 3’UTR suppresses breast cancer stemness by inhibiting YAP/TAZ activity through co-regulating Hippo and RhoA (Ras homolog gene family, member A) signaling [23]. Notably, recent work demonstrates that miR-125b promotes liver fibrosis and hepatic stellate cell activation by the activation of RhoA signaling [43], and our results also demonstrate that RhoA and Hippo signaling are involved in Tanshinone IIA-mediated effects on breast cancer cell stemness (data not shown, **GSE156155**). Thus, we assume that this miR-125b/STARD13 axis enhances breast cancer stemness probably through RhoA and Hippo signaling, which should be further investigated.

As the carcinogenic roles of miR-125b in the progression of breast cancer, we aim to find potential drugs that could inhibit miR-125b activity. The previous studies have shown Tanshinone IIA effects on regulating miRNAs activity [8, 44, 45] and Tanshinone IIA could inhibit the migratory and invasive ability of cervix carcinoma stem cells via inhibition of YAP transcriptional activity that is also suppressed by STARD13 3'UTR [11]; these effects promote us to assess whether Tanshinone IIA could attenuate miR-125b activity in breast cancer cells. As expected, based on the data analysis of miRNAs-based transcriptome-sequencing, we found that Tanshinone IIA downregulated miR-125b level and attenuated its inhibitory effects on STARD13 expression. Additionally, miR-125b overexpression partially rescued the suppression of Tanshinone IIA on breast cancer stemness; these effects prompt that miR-125b is a target of Tanshinone IIA in breast cancer cells. However, as shown in Fig. 4A, G, H a significant change in STARD13 expression is observed before a significant change in miR-125b (ie at 2.5 μM dose), we assumed that other miRNAs could be involved in Tanshinone IIA-mediated regulation on STARD13 expression as denoted in Fig. 3G, such as miR-9 and miR-10b that had been previously confirmed to target STARD13 as well by our group [21, 23], and miR-146a, miR-210, miR-25, miR-103 and miR-484 have been shown to promote tumor stemness [18, 46–50]. In addition, the previous study has shown that Tanshinone IIA enhances doxorubicin sensitivity of breast cancer by decreasing the expression of MDR-related ABC transporters [51], as CSCs hold the higher expression levels of MDR-related ABC transporters compared to that in cancer cells [52], this effect further supports our results that Tanshinone IIA could attenuate breast cancer stemness. Consistently, the pathway of *ABC transporters* is enriched in Tanshinone IIA-treated breast cancer cells (Fig. 3B). Notably, our results are consistent with the results presented in the previous study [53], in which the dependent mechanism by which Tanshinone IIA exerts its effects was not shown and just one cell line was used. Thus, our work is a good complement to the previous studies. Furthermore, it must be noted that Tanshinone IIA held no effect on the cell cycle progression of MCF-7 cells and viability of MCF-10A cells, while it arrested cell cycle in G1 phase in MDA-MB-231 cells (Additional file 1: Figure S1), which means MDA-MB-231 cells are more sensitive to Tanshinone IIA than MCF-7 cells, this could be due to the higher stemness of MDA-MB-231 cells compared to that of MCF-7 cells and subsequently predicts that Tanshinone IIA holds a stronger inhibition on breast CSCs than that of breast cancer cells. Besides, MTT analysis showed that Tanshinone IIA displayed a little effect on MDA-MB-231 cell viability, this seems to be inconsistent to the cell cycle results; However, we speculate that this discrepancy might be resulted by the different sensitivity of MTT and flow cytometric assay.

Finally, as CSCs could lead to chemoresistance and adriamycin is the first-line drug for breast cancer treatment, the effects of Tanshinone IIA on adriamycin sensitivity and resistance were evaluated, and it was identified that Tanshinone IIA positively regulated adriamycin sensitivity. Notably, Tanshinone IIA could also reduce MCF-7-Adr cell stemness. Our results demonstrate that Tanshinone IIA could be combined with adriamycin in breast cancer treatment.

## Conclusion

Currently, we demonstrate that the inhibition on the miR-125b/STARD13 axis mediated by Tanshinone IIA attenuates breast cancer stemness and adriamycin sensitivity, providing new clues for breast cancer treatment.

## Supplementary Information

**Additional file 1: Figure S1.** The effects of Tanshinone IIA on the cell cycle distribution of breast cancer cells and viability of MCF-10A cells. **A**, **B** The effects of Tanshinone IIA on the cell cycle distribution were determined in MCF-7 (**A**) and MDA-MB-231 (**B**) cells with different concentrations. **C** MCF-10A cells were treated with different concentrations of Tanshinone IIA and subjected to cell viability assay using MTT method. **D** EdU incorporation analysis was performed in tumors pre-treated with Tanshinone IIA or not. **p < 0.01 vs. Control, n = 6.

**Additional file 2: Figure S2.** MiR125b inhibition attenuates MDA-MB-231 cell stemness dependent on STARD13. **A** MDA-MB-231 cells with a transfection of miR-125b inhibitor as well as si-STARD13 or not were subjected to the examination of stemness markers (Oct4 and ALDH1A1) mRNA levels. **B** Stemness markers (Oct3/4 and ALDH1A1) protein levels were detected in **A**-depicted cells. **C** ALDH activtiy was measured in **A**-depicted cells. **D** Representative FACS profile of **A**-depicted cells were shown with CD44^+^  and CD24^−^ markers. **E**, **F** Spheroid formation ability was evaluated in **A**-depicted cells. **p < 0.01 vs. Control, ^##^p < 0. 01 vs. miR-125b inhibitor, n = 3. For transfection experiments, control groups were transfected with miR-125b inhibitor NC.

**Additional file 3: Figure S3.** Tanshinone IIA reduced stemness marker expression dependent on miR-125b in vivo. **A** IHC analysis on stemness markers expression in tumors derived from cells treated as indicated. **B** The IHC results denoted in **A** were quantified via quantity one software.

**Additional file 4: Figure S4.** Tanshinone IIA attenuates MCF-7-Adr cell stemness. **A**, **B** MCF-7-Adr cells were treated with different concentrations of Tanshinone IIA, and followed by examinating stemness marker expression by qRT-PCR (**A**) and western blot (**B**). **C**, **D** Capacity of spheroid formation was detected in Tanshinone IIA-treated MCF-7-Adr cells. **E** ALDH activity was measured in **C**-depicted cells (**C**). **F** Representative FACS profile of **A**-depicted cells were shown with CD24^−^ and CD44^ +^ markers. n = 3, **p < 0.01 vs. Control.

## Data Availability

All data generated or analyzed during this study are included in this published article and its supplementary information files.

## Abbreviations

CSCs: Cancer stem cells
VEGF: Vascular endothelial growth factor
VEGFR2: Vascular Endothelial Growth Factor Receptor 2
YAP: Yes-associated protein
MiRNAs: MicroRNAs
3'UTR: 3' Untranslated region
STARD13: StAR-related lipid transfer domain protein 13
TAZ: Transcriptional coactivator with PDZ-binding motif
STR: Short tandem repeat
FBS: Fetal bovine serum
QRT-PCR: Quantitative Real-time PCR
cDNA: Complementary DNA
snRNA: Small nuclear RNA
Oct3/4: Octamer-binding transcription factor 4
PARP: Poly ADP-ribose polymerase
NC: Negative control
si-STARD13: SiRNA against STARD13
MTT: 3-(4,5-Dimethyl-2-thiazolyl)-2,5-diphenyl-2-H-tetrazolium bromide
GEO: Gene Expression Omnibus
IHC: Immunohistochemistry
SD: Standard deviation
GO: Gene Ontology
KEGG: Kyoto Encyclopedia of Genes and Genomes
TPM: Transcripts PerKilobase Million
TNBC: Triple negative breast cancer
MKL1: Myocardin-Like Protein 1
IL11: Interleukin11
MAPK: Mitogen-activated protein kinases
STAT3: Signal transducer and activator of transcription 3
RhoA: Ras homolog gene family, member A
