# Supplementary figures and images for: Tanshinone IIA attenuates the stemness of breast cancer cells via targeting the miR-125b/STARD13 axis
Source: Exp Hematol Oncol. 2022 Jan 20;11:2. doi: 10.1186/s40164-022-00255-4 (PMC8781032; doi:10.1186/s40164-022-00255-4)

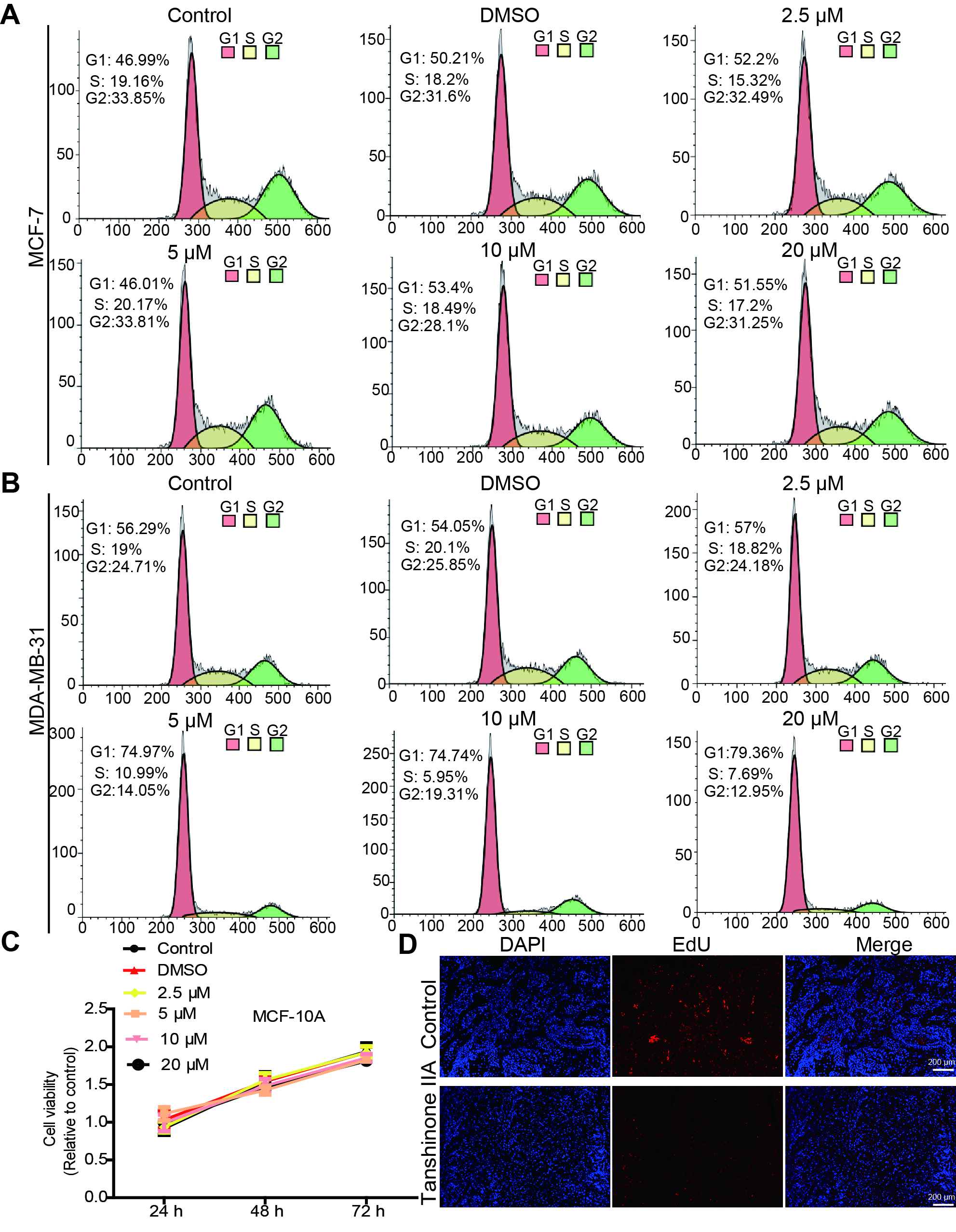

Supplement: Supplementary file 1 — Additional file 1: Figure S1. The effects of Tanshinone IIA on the cell cycle distribution of breast cancer cells and viability of MCF-10A cells. A, B The effects of Tanshinone IIA on the cell cycle distribution were determined in MCF-7 (A) and MDA-MB-231 (B) cells with different concentrations. C MCF-10A cells were treated with different concentrations of Tanshinone IIA and subjected to cell viability assay using MTT method. D EdU incorporation analysis was performed in tumors pre-treated with Tanshinone IIA or not. **p < 0.01 vs. Control, n = 6. [file 40164_2022_255_MOESM1_ESM.tif]

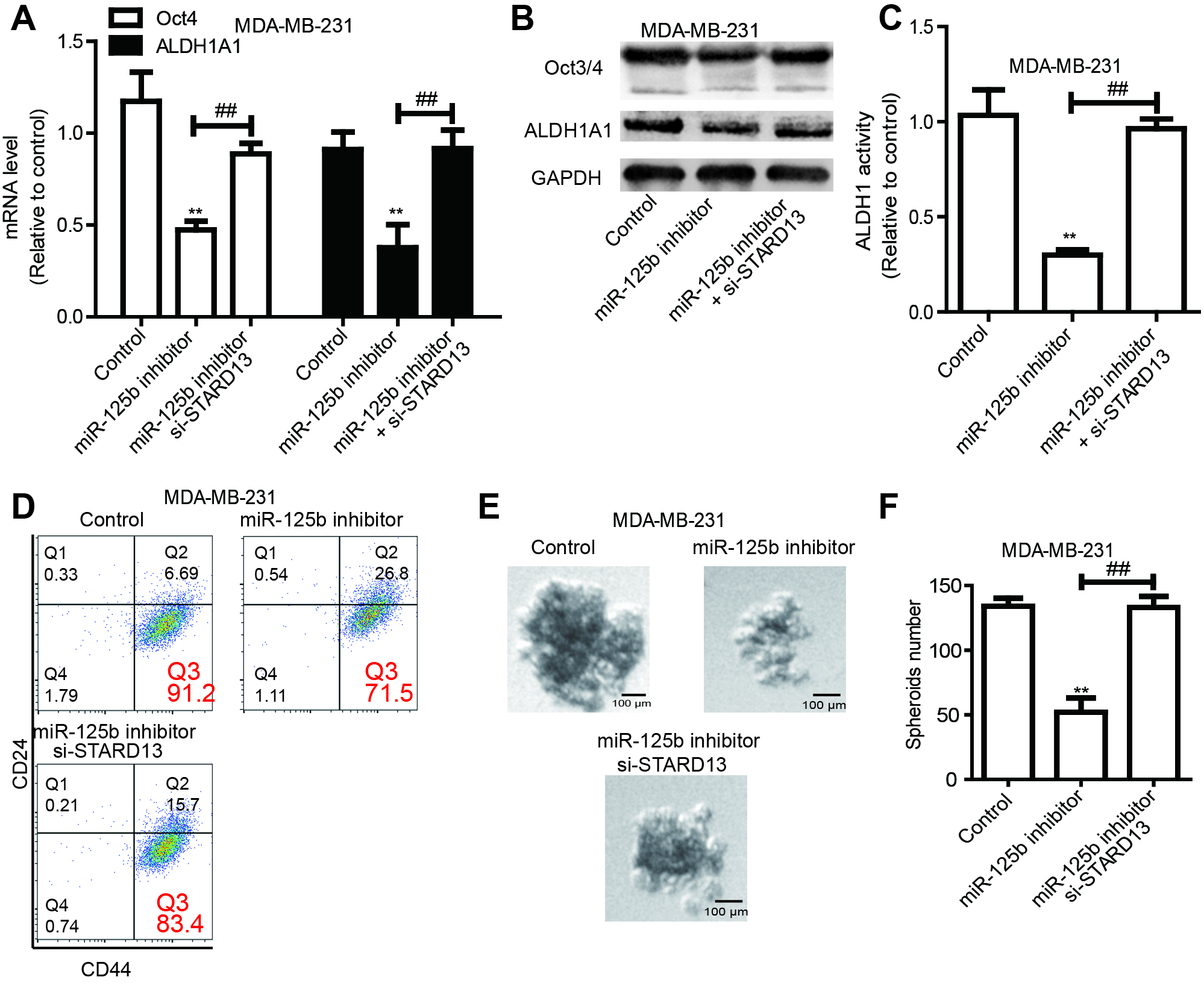

Supplement: Supplementary file 2 — Additional file 2: Figure S2. MiR125b inhibition attenuates MDA-MB-231 cell stemness dependent on STARD13. A MDA-MB-231 cells with a transfection of miR-125b inhibitor as well as si-STARD13 or not were subjected to the examination of stemness markers (Oct4 and ALDH1A1) mRNA levels. B Stemness markers (Oct3/4 and ALDH1A1) protein levels were detected in A-depicted cells. C ALDH activtiy was measured in A-depicted cells. D Representative FACS profile of A-depicted cells were shown with CD44+ and CD24− markers. E, F Spheroid formation ability was evaluated in A-depicted cells. **p < 0.01 vs. Control, ##p < 0. 01 vs. miR-125b inhibitor, n = 3. For transfection experiments, control groups were transfected with miR-125b inhibitor NC. [file 40164_2022_255_MOESM2_ESM.tif]

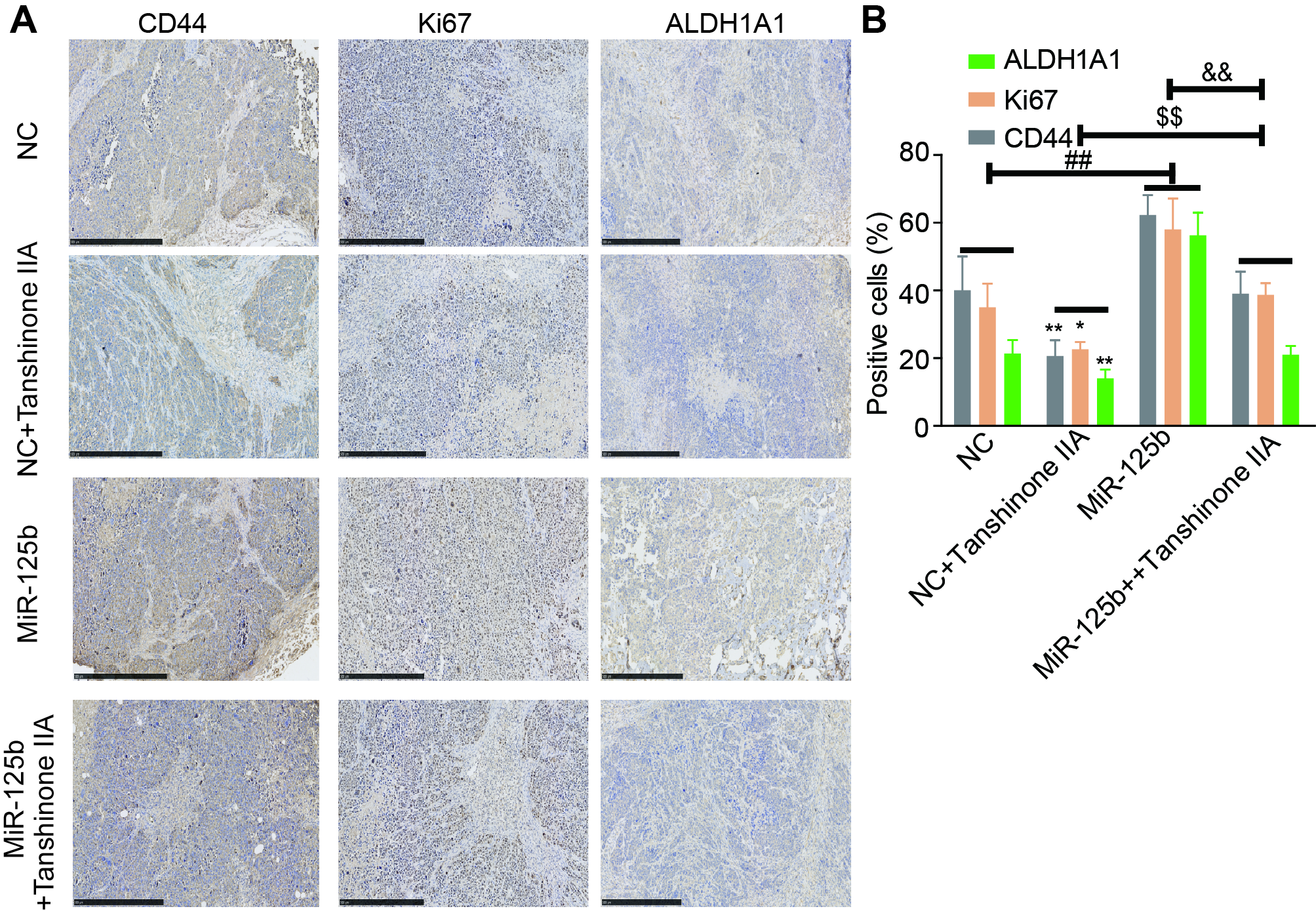

Supplement: Supplementary file 3 — Additional file 3: Figure S3. Tanshinone IIA reduced stemness marker expression dependent on miR-125b in vivo. A IHC analysis on stemness markers expression in tumors derived from cells treated as indicated. B The IHC results denoted in A were quantified via quantity one software. [file 40164_2022_255_MOESM3_ESM.tif]

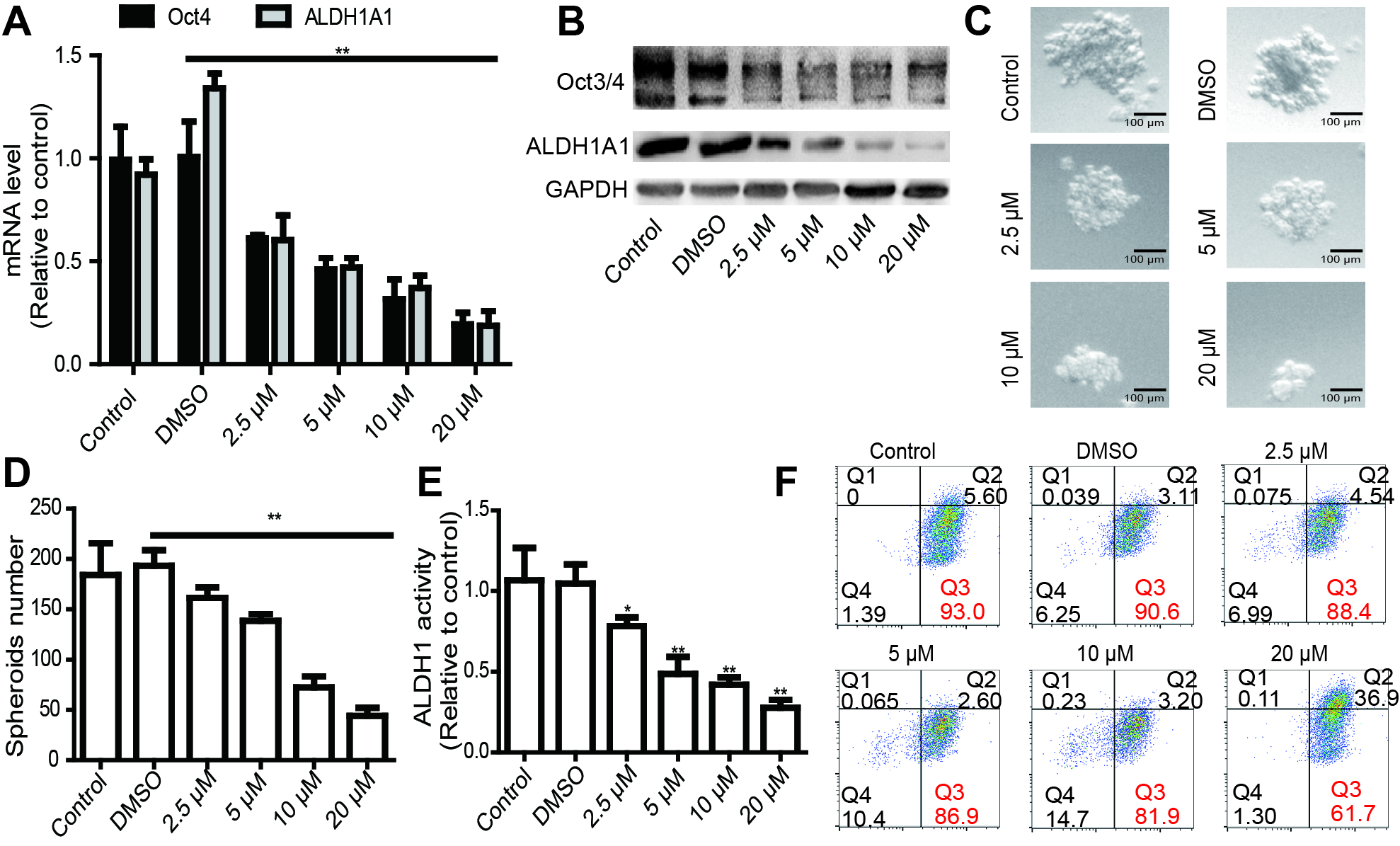

Supplement: Supplementary file 4 — Additional file 4: Figure S4. Tanshinone IIA attenuates MCF-7-Adr cell stemness. A, B MCF-7-Adr cells were treated with different concentrations of Tanshinone IIA, and followed by examinating stemness marker expression by qRT-PCR (A) and western blot (B). C, D Capacity of spheroid formation was detected in Tanshinone IIA-treated MCF-7-Adr cells. E ALDH activity was measured in C-depicted cells (C). F Representative FACS profile of A-depicted cells were shown with CD24− and CD44 + markers. n = 3, **p < 0.01 vs. Control. [file 40164_2022_255_MOESM4_ESM.tif]
